# The Common Threads in Program Evaluation

**Published:** 2005-12-15

**Authors:** William R Shadish

**Affiliations:** School of Social Sciences, Humanities, and Arts, University of California at Merced

A well-known evaluator once said, "Evaluation — more than any science — is what people say it is, and people currently are saying it is many different things" (1). Ask an economist what program evaluation is, and you will get a very different answer than if you asked a psychologist; and they would both differ from what an educator might say. Indeed, the field is so large and diverse, and the use of the term *program evaluation* is so ubiquitous, that it is often difficult to discern any common threads. Yet common threads do exist, and I would like to point to some of them in the articles of this special issue of *Preventing Chronic Disease*.

Five common concerns are woven throughout the literature on program evaluation (2). First is a concern with *how to construct valid knowledge*. This concern has both a philosophical component and a methodological component; the philosophical component concerns the kinds of things we can know about programs, and the methodological component concerns the designs, measures, and analyses that we use to create and organize data. Second is a concern with *how we place value* on evaluation results. One often hears it said that the data speak for themselves, but that is rarely the case. This concern articulates the many theoretical and practical tools we have available to help us in this valuation. Third is a concern with *how programs change*. After all, program evaluation is intended to be a very practical area of study, one that aims to make a real difference in people's lives. If we do not know the leverage points for program change, we cannot apply evaluation results to gain that leverage. Fourth is *how to use evaluation results* in the policy process. This concern is about how to get our results to the stakeholders who influence those leverage points in a way that helps stakeholders make use of the data. Fifth and finally is the paramount concern with *how to organize evaluation practice*, given the implications of all the preceding issues for what the evaluator actually does in any given evaluation. This fifth concern is always a matter of tradeoffs, for one can never do everything well in a single evaluation.

## Knowledge Construction

In the early years of program evaluation, evaluators approached the task using the methods they learned in their primary disciplines. Psychologists tended to use experiments, educators relied heavily on measurement, and economists leaned toward sophisticated statistical analysis of observational data. Gradually, however, evaluators realized that no single method was enough. The choice of evaluation method should follow question choice, but so many different questions are important in program evaluation that no single method can answer all of them well. As examples, evaluators are asked to answer questions about the following:

- ***Need.*** Every program targets a real or perceived need, such as the need to reduce rates of HIV infection, hypertension, or diabetes or to reduce the costs associated with the care of those conditions. Characterizing those needs requires such methods as needs assessment, stakeholder surveys, and epidemiological studies of incidence and prevalence. Jack et al (3) describe how the National Center for Chronic Disease Prevention and Health Promotion of the Centers for Disease Control and Prevention uses public health surveillance and epidemiologic studies to measure the need for behavioral interventions.
- ***Program implementation.*** Programs can be large enterprises composed of multiple elements that come into play to different degrees over time. Creating a program infrastructure is an early task, engaging clients and providers comes a bit later, and providing follow-up care comes even later. Assessing program implementation requires such methods as inspection of records, cost analysis, creation of management information systems, and observation of client interactions with the program from intake to follow-up, with all these methods being differentially useful depending on the stage of program implementation. Besculides et al (4) use a combination of quantitative measures and qualitative interviews to assess successful implementation of lifestyle programs for women.
- ***Program outcome.*** A key rationale for implementing programs is that they will result in beneficial outcomes. The most precise method for measuring those outcomes is often a randomized experiment. When randomization is not feasible for ethical or practical reasons, or the precision yielded by randomization is unnecessary, many kinds of nonrandomized experiments can be used, such as interrupted time series, regression discontinuity, and nonequivalent comparison group designs. Sometimes the interest is not in attributing outcomes to the intervention but in monitoring outcomes over time to see if they are approaching a defined standard, as with the study by Mukhtar et al (5) of progress toward meeting selected national health outcomes in diabetes.

No single method can provide a precise answer to all of these questions. That is why the organization of evaluation teams benefits so much from being multidisciplinary.

Not surprisingly, then, the articles in this special issue describe a wide array of evaluation methods because the authors are asking many different kinds of questions. Among the methods and tools used are focus groups (4,6), logic models (7), and monitoring of program outcomes (5). Some studies used a combination of methods, sometimes to answer more than one question and sometimes to try to capture the merits of both high bandwidth and high fidelity in one study. Besculides and colleagues (4) did this in their study of best practices in implementing lifestyle interventions targeting women. So did Houston et al (6) in their study of a 1-day lay health diabetes conference and Tucker et al (7) in their evaluation of the REACH 2010 initiative. The cost of such mixed-methods research is that one must take away from Peter to pay Paul; because evaluation budgets are always limited, we can devote fewer resources to doing one method well if we spread those resources over more than one method. Whether or not this tradeoff is acceptable is a decision that must be made on a case-by-case basis.

## Valuing

Value judgments are present throughout an evaluation. One example is choosing outcome variables. Outcomes connect to values in two ways. The first is that some outcomes are measures of the need to which the program is intended to respond, so that a program is better to the extent that it ameliorates those needs. For example, if we cite the fiscal costs of chronic disease as the need that justifies a public health intervention, then that intervention ought to reduce those costs. An example is in the study by Rein et al (8), who argue that interventions to prevent hypertension should both improve health and reduce costs. The second way that outcomes connect to values is through stakeholder opinions. Programs have stakeholders whose opinions vary widely about what is a good outcome. For instance, one study of the outcomes of long-term care for people who were chronically mentally ill (9) found that patients and their families valued safe shelter, good food, and adequate medical care, but federal and academic stakeholders valued programs that helped patients move toward independent living. Especially when achieving one outcome sometimes comes at the cost of sacrificing another, stakeholders often disagree about whether an outcome is good or bad. This is one reason why so many approaches to evaluation start with identification of and contact with program stakeholders, as in Martin and Heath's (10) use of a six-step model that starts with engaging stakeholders.

Evaluators also deal with value judgments when they (sometimes implicitly) set standards by which they judge how much improvement is sufficient to be valuable, what is sometimes called *practical significance*. A common implicit standard is to compare the treatment to a control, declaring the treatment good if it improves upon what is accomplished by the control. An innovation is often thought to be especially valuable if it improves on the outcomes of a usual treatment control, as opposed to a no-treatment control. Rein et al (8) provide an example when they compare health outcomes under a state-funded education and direct service program with both no preventive treatment for high blood pressure and private-sector preventive treatment. Most stakeholders would argue that a public intervention that improves over private-sector treatment is more valuable than one that merely improves over no treatment at all. However, evaluators sometimes also refer to minimum absolute standards, and if a program falls below this level, it fails no matter how it performs in other respects. For example, Mukhtar et al (5) use the *Healthy People 2010* objectives for selected diabetes outcomes as a standard that must be met to reach a positive evaluation. Such absolute cutoffs tend to be rare, and even when they are available they tend to be used in combination with comparison to a control.

Finally, evaluators deal with value judgments when they synthesize the diverse results of a study to reach an overall evaluative conclusion. Because stakeholders value different outcomes differently, a single overall synthesis is often difficult to justify to all parties. For example, the tradeoff between lowering one's blood pressure and risking sexual impotence may be valued differently by the researcher and the patient, as witnessed by the number of therapies that researchers may judge successful but with which some patients refuse to comply. Consider, for instance, the evaluation of *Sesame Street* by Bogatz and Ball (11). It found that children exposed to *Sesame Street* learned several more letters of the alphabet per year than did control group children but that disadvantaged children learned less than advantaged children. Is that good or bad? Cook and his colleagues (12) argued that if you believe that such programs should be improving outcomes on average, then it is good; but if you believe such programs should be closing the gap between the most and least needy children, then it is bad. The most common safeguard is to seek diverse input from stakeholders about how they prioritize among the results and then refer to those priorities by creating multiple syntheses that reflect the major positions among stakeholders (13).

## Social Programs

The role of program evaluator is not the same as the role of program developer. Indeed, some evaluators argue that the two roles are incompatible because the developer is often biased toward wanting a positive evaluation of the program (14). Still, many evaluators find themselves involved in program development because they often have broad experience that comes from having evaluated similar kinds of programs in the past, because they know that it often makes for a better evaluation if the evaluator can assist with program development from the start, or because their job description calls for both activities. Balamurugan and colleagues (15) illustrate this melding of the two roles in their article about programs for diabetes self-education management. They show how the lack of advanced evaluation planning impeded not only the evaluation but also the effectiveness of the program itself.

All evaluators benefit from knowledge of how programs come into being, change, end, and function in their environment. For example, if we aim to create sustainable public health programs, we must know the economic, social, political, and psychological factors that make programs sustainable. Similarly, if we believe that individual, family, health system, community, and societal factors all contribute to the rise of chronic disease (3), then we have to do research on those factors to know how to change them. For example, Besculides et al (4) study factors that lead to successful lifestyle interventions targeting women; such knowledge tells the evaluator where the leverage points for productive program change might be so that the evaluation can be directed toward answering questions about those points.

A general rule of thumb is that the smaller the intervention, the more easily it can be eliminated from or added to the things that service providers do — which makes change more feasible. For example, if Houston et al's (6) 1-day lay health diabetes conference is effective, it is easy to disseminate it to other places; and if it is ineffective, it could be terminated and the resources moved elsewhere without too much resistance. This is far less the case for larger interventions, such as an entire clinic devoted to health promotion. Starting such clinics in other places is an expensive and time-consuming endeavor, and closing down such clinics entirely is a rare and often controversial event. Another rule of thumb is that the kinds of evaluation activities we use with new programs should be different from those we use with mature programs. For new programs, we should use evaluation activities associated with needs assessment and program implementation. For mature programs, we should emphasize outcome evaluation — after the program has worked out the initial kinks that inevitably occur when a program begins.

## Use of Results in Policy

Maximizing the chances that evaluation results will be used is a paramount concern in evaluation. In this special issue, use of evaluation results is discussed explicitly by Martin and Heath (10) as part of a six-step model of evaluation. In the early years, few evaluators thought much about whether evaluation results would be used in policy. They simply assumed that their results would be used once presented, but having evaluation results used proved complicated. First, several kinds of use can occur. These include *instrumental use* in which evaluation results are used to make a policy decision, *conceptual use* in which evaluation results may change the way stakeholders think about policy even though the results may not result in an immediate policy change, and *persuasive use* in which evaluation results are used to advocate for or against a policy. Instrumental use tends to occur least frequently and often involves small changes, because small changes are often more feasible than big changes. Conceptual use is ubiquitous among those who keep informed about a policy issue and can have a profound impact over time on how future generations of stakeholders shape the policy process. Persuasive use is also common from lobbyists to legislators who desire certain policies to be established and who use evaluation results to support their case.

Second, use can occur at any time after evaluation results are presented. Some use occurs immediately, but much use occurs later, sometimes decades later (16). The more immediate the use, the greater the likelihood that the change made is a small and incremental one. Large changes to a system take time because they involve so many ancillary changes and because changes that are not immediately feasible often become feasible later when the context has changed.

Third, use rarely happens without the evaluator doing things to make it happen. Evaluators have learned that instrumental use can be facilitated by having frequent and early contact with users, studying things the user actually controls, clarifying action implications of findings, and disseminating results in forms other than traditional research reports. Conceptual use can be facilitated by challenging fundamental ideas and assumptions and by circulating results throughout the network of people concerned with the issues in the outlets they read.

## Evaluation Practice

All of the prior issues come together in evaluation practice, in which evaluators must decide whether to do an evaluation, what questions to ask and methods to use, how to involve stakeholders in the evaluation, what values should be represented, and how to facilitate use. After all, time and resource constraints imply that evaluators cannot, for example, ask every question, use every method, or foster every kind of use. Many evaluators therefore use a set of concepts that help them to focus their practice. For example, Jack et al (3) note that "*decision and accountability*, *utilizations focused*, *client centered and responsive*, *case study*, and *outcomes monitoring and value added* are a few examples of evaluation approaches." In addition, Lavinghouze (17) describes the theory-driven approach to evaluation. Such evaluation approaches are ways of helping the evaluator decide on the tradeoffs involved in conducting an evaluation.

Evaluation practice also entails often-complex structures for how the evaluation process is to be organized. Even the lone evaluator within a health center faces organizational obstacles to evaluation. As the evaluation context grows, the organizational challenges increase dramatically. Discussions of this issue figure prominently in several  articles in this issue of *Preventing Chronic Disease*, including MacDonald et al's (18) description of methods for coordinating national and community-level evaluation efforts in the Steps to a HealthierUS program, Balamurugan et al's (15) discussion of ways to organize evaluation in rural Arkansas, and Tucker et al's (7) analysis of how to combine local site-specific evaluations with national evaluations that are able to synthesize at least some of the evidence across sites.

## Conclusion

Public health has a long history of involvement in program evaluation. Indeed, many evaluators have forgotten that the first textbook on program evaluation in public health, by Edward Suchman (19), was published in 1967. The field has made much progress since then (2,20), and it is a pleasure to see this tradition continued in the articles of this issue.

## References

[1] Glass GV, Ellett FS (1980). Evaluation Research. Annu Rev Psychol.

[2] Shadish WR, Cook TD, Leviton LC (1991). Foundations of program evaluation: theories of practice.

[3] Jack L, Mukhtar Q, Martin M, Rivera M, Lavinghouze R, Jernigan J (2006). Program evaluation and chronic diseases: methods, approaches, and implications for public health. Prev Chronic Dis.

[4] Besculides M, Hesketh H, Farris R, Will J (2006). Identifying best practices for WISEWOMAN programs using a mixed-methods evaluation. Prev Chronic Dis.

[5] Mukhtar Q, Jack L, Murphy D, Martin M, Rivera MD (2006). Evaluating progress toward Healthy People 2010 national diabetes objectives. Prev Chronic Dis.

[6] Houston J, Williams J, Martin M, Hill R (2006). The Annual African American Conference on Diabetes: evolving program evaluation with evolving program implementation. Prev Chronic Dis.

[7] Tucker P, Liao Y, Giles WH, Liburd L (2006). The REACH 2010 logic model: an illustration of expected performance. Prev Chronic Dis.

[8] Rein D, Orenstein RC, Chen H, Jones P, Brownstein N, Farris R (2006). A cost evaluation of the Georgia Stroke and Heart Attack Prevention Program. Prev Chronic Dis.

[9] Shadish WR, Thomas S, Bootzin RR (1982). Criteria for success in deinstitutionalization: perceptions of nursing homes by different interest groups. Am J Community Psychol.

[10] Martin S, Heath G (2006). A six-step model for evaluation of community-based physical activity programs. Prev Chronic Dis.

[11] Bogatz GA, Ball S (1971). The second year of Sesame Street: a continuing evaluation, Volumes 1 and 2.

[12] Cook TD, Appleton H, Conner RF, Shaffer A, Tamkin G, Weber SJ (1975). "Sesame Street" Revisited.

[13] Shadish WR, Leviton LC, Benson AP, Hinn DM, Lloyd C (2001). Descriptive values and social justice. Visions of quality: how evaluators define, understand, and represent program quality.

[14] Scriven M (1976). Evaluation bias and its control. Evaluation Studies Review Annual.

[15] Balamurugan A, Rivera M, Jack Jr, Allen K, Morris S (2006). Barriers to diabetes self-management education programs in underserved rural Arkansas: implications for program evaluation. Prev Chronic Dis.

[16] Polsby NW (1984). Political innovation in American: the politics of policy initiation.

[17] Lavinghouze RS (2006). Practical program evaluation: assessing and improving planning, implementation, and effectiveness [book review]. Prev Chronic Dis.

[18] MacDonald G, Garcia D, Zaza S, Compton D, Schooley M (2006). Steps to a HealthierUS Cooperative Agreement Program: foundational elements for program evaluation planning, implementation, and use of findings. Prev Chronic Dis.

[19] Suchman EA (1967). Evaluative research: principles and practice in public service and social action programs.

[20] Rossi PH, Lipsey MW, Freeman HE (2003). Evaluation: a systematic approach.

